# Bridging the Gap: Developing a Co‐Designed Resource to Support the Hospital‐to‐Home Transition Following Limb Loss

**DOI:** 10.1111/hex.70825

**Published:** 2026-08-17

**Authors:** Ciara Everard, Melissa C. Day, Ross Wadey, Kimberley Humphrey

**Affiliations:** ^1^ School of Medicine Trinity College Dublin Dublin Ireland; ^2^ Institute of Sport University of Chichester Chichester UK; ^3^ Faculty of Sport, Technology, and Health Sciences St. Mary's University London UK

**Keywords:** co‐design, co‐production, limb absence, qualitative

## Abstract

**Background:**

During the hospital‐to‐home transition, people with limb loss and their caregivers can face several practical, physical, social and psychological challenges. These challenges are amplified by gaps in transitional care support, ultimately leading to readmissions and increased healthcare costs.

**Objective:**

This co‐design research project aimed to help bridge the gap between hospital to home by working collaboratively with 10 healthcare providers, 14 people with limb loss, and 15 caregivers to (a) understand the transition from hospital to home and identify gaps in transitional care; and (b) co‐construct a resource that aimed to bridge these gaps and better facilitate this transitionary process.

**Methods:**

Underpinned and informed by experience‐based co‐design, ethnographic observation (*n* = 18 months) and semi‐structured interviews (*n* = 49) were used to gather and understand service‐users, healthcare providers, and caregivers' experiences of the transition and identify support gaps. Three working groups were then formed to co‐design a resource to address support gaps.

**Results:**

Four gaps in transitional support were identified: information support, communication support, emotional and social support, and care continuity. Recommendations for addressing these gaps were developed with people with limb loss and their caregivers and disseminated to staff members within the rehabilitation centre. A co‐designed educational booklet was then created that aimed to address all four support gaps.

**Conclusion:**

Overall, this study aims to guide future researchers and limb centre rehabilitation teams in addressing gaps in transitional care by centralising the voices of service‐users and caregivers, ensuring that they shape the development of evidence‐based strategies for meaningful and sustainable change.

**Patient and Public Contribution:**

Health care workers, people with limb loss, and their caregivers were actively involved throughout the study, contributing to both its design and development. Their insights played a central role in identifying support gaps, refining study priorities, and developing actionable recommendations. In addition, they participated in co‐design workshops where they collaboratively co‐created the educational resource to ensure it was relevant, accessible, and responsive to their lived experiences.

## 1 Bridging the Gap: Developing a Co‐Designed Resource to Support the Hospital‐to‐Home Transition Following Limb Loss

Amputation is a major life‐changing experience that involves not only physical adjustment to the loss of a body part, but a psychosocial adjustment as well [1]. In adapting to life after an amputation, previous research has highlighted how the hospital‐to‐home transition can be particularly acute, as service‐users and their caregivers negotiate significant practical, physical, psychological, and social challenges [2]. For example, practical and social challenges often include adapting or finding a new home, re‐negotiating current employment, and adjusting to new roles within the household. On a physical level, people with limb loss may struggle with navigating prosthesis use, impaired balance, reduced mobility, and increased energy expenditure [3]. These everyday practical, physical, and social challenges often coincide with and create psychological challenges, as this liminal period, in transitioning from the hospital to home, has been reported to be marked with isolation, chaos, and confusion [4, 5].

In recognising these challenges, researchers have highlighted how preparing service‐users for life post‐amputation and providing psycho‐educational resources (e.g., educational talks, booklets and access to support services) during rehabilitation are key in helping to prime service‐users and their caregivers for the journey ahead [5]. To facilitate this transition, amputee rehabilitation centres advocate for a collaborative person‐centred rehabilitation approach that aims to optimise mobility, function, and independence, equipping service‐users and caregivers with the tools to rebuild their lives in ways that are meaningful and bespoke to them [6]. Despite these efforts, gaps in transitional support still arise, and deficits in care pathways continue to persist. For example, previous research has highlighted that hospital staff (e.g., physiotherapists, occupational therapists) often lack the time, resources, or community services to adequately support service‐users in managing the hospital‐to‐home transition [3, 7, 8]. Moreover, service‐users have reported a lack of informational and psychological support, insufficient integration of meaningful activities into rehabilitation to aid skill transfer post‐discharge, and limited resources for managing day‐to‐day tasks [9]. Collectively, these shortcomings contribute to service‐users feeling unprepared for the transition home. In addition to service‐users' concerns, research has highlighted how gaps in communication between hospital staff and caregivers leave them feeling ill‐equipped to fulfil their responsibilities [10]. Ultimately, this inadequate transitional care can lead to poor self‐management, readmissions, and increased healthcare costs [11, 12].

Aligned with this evidence‐based rationale, as a research team, we were approached by a service provider (i.e., National Health Centre (NHS) limb centre in England) who identified a need to better facilitate the hospital‐to‐home transition. The service provider engaged in a period of consultations between healthcare practitioners (HCP) and service‐users, whereby the difficulties of the hospital‐to‐home transition were discussed. Additionally, it was highlighted that, although caregivers impact and were impacted by this transitional period, they were often the forgotten voice within rehabilitation services. Consequently, the service providers invited us, as academic researchers in (i.e., outside‐in pathway; 13), to form a working group with them. The purpose of this commissioned research would be to help bridge the gap between hospital and home for people with limb loss, by working collaboratively with service‐users, caregivers, and HCPs within this amputee rehabilitation centre.

Following several discussions, a co‐design methodology was chosen, as it acts to foreground service‐users' experiences, which dovetailed with the person‐centred ethos of the limb centre and NHS [13]. Moreover, co‐design is a participatory research methodology, and hence it promotes a collaborative approach by bringing together researchers, service users, and service providers to identify and implement improvements in care [13]. This participatory element gives primacy to the right of people to actively shape the worlds in which they act, thus creating change in ways that are meaningful and relevant to them [13]. To this end, co‐design methodologies have been advocated to help reduce redundancies often seen in health research where interventions are created that are deemed to be impractical or irrelevant to specific groups. Indeed, co‐design is said to be particularly valuable in creating patient‐centred research that holds real‐world applicability and therefore is likely to integrate seamlessly into clinical practice [14].

Evidence of the effectiveness of co‐design methodologies has coalesced with its increased use [15]. In particular, previous research has illustrated how co‐design can enhance transitional care, addressing communication gaps by fostering more person‐ and family‐centred approaches, and ultimately enhancing health outcomes and quality of life [16]. That said, despite the proven benefits of co‐design and the aforementioned gaps in transitional care for people with limb loss, co‐design has yet to be employed within a limb loss context. Therefore, the aim of this study was to help bridge the gap between hospital and home by using a co‐design methodology to work collaboratively with HCPs, service‐users, and caregivers within a limb centre. Overall, this paper aims to make an empirical and practical contribution to the literature by outlining how the stages of a co‐design methodology were used to: (a) understand the transition from hospital to home and identify gaps in transitional care; and (b) co‐construct a resource that aims to bridge these gaps and better facilitate the transitionary process.

## 2 Methods

### 2.1 Methodology

Experience‐based co‐design (EBCD) was the type of co‐design methodology chosen for the study. EBCD is similar to other participatory‐based approaches in that it aims to engage a variety of stakeholders to collaboratively identify and implement service improvements. However, what distinguishes EBCD from other co‐design approaches is its focus on people's experiences as a motivator for change, which are captured and conveyed through the medium of storytelling [17]. The EBCD process [18, 19] is composed of six stages and involves: (1) Setting up the project; (2) gathering staff experiences through observation and in‐depth interviews; (3) gathering service‐user and caregiver experiences through observation and filmed narrative‐based interviews; (4) bringing staff, service‐users, and caregivers together to share their experiences of a service and identify their shared priorities for improvement. This event is said to be prompted by an edited 30 min “trigger” film of service‐user narratives, where touchpoints (critical moments experienced in relation to the service) are discussed and prioritised; (5) once priorities are identified, small groups of service‐users and staff are formed to co‐design improvements to the service; and 6) finally, a celebration event is held to review progress made [17].

**Figure 1:**
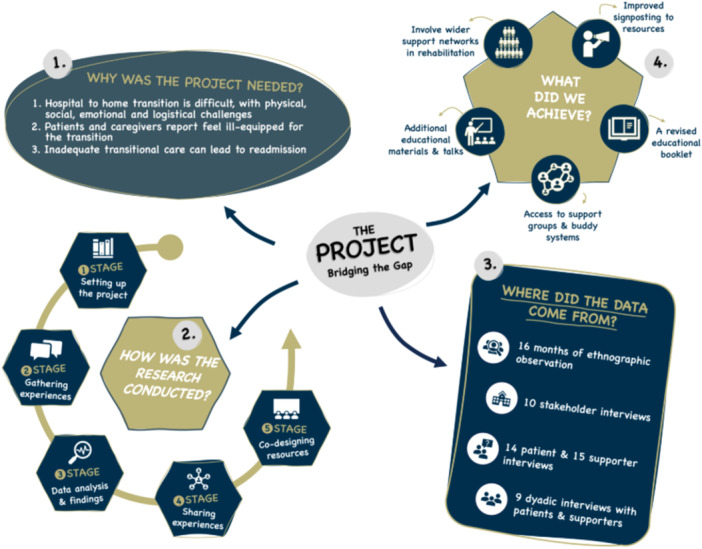
Overview of the process.

Aligned with recent researchers who have advocated for the need for adapted forms of EBCD, especially when working with vulnerable populations [20], both the rehabilitation and research team decided to use a modified form of EBCD due to ethical (e.g., anonymity), and logistical reasons (e.g., staff and service‐user workload). For example, although the EBCD design recommends that a trigger‐based, filmed narrative of service‐user interviews we decided to disseminate narratives of service‐users' and caregivers' experiences in an audio format (using voice‐overs) to help safeguard their confidentiality and anonymity, in accordance with our NHS and institutional ethical approval requirements. Additionally, because limb loss service‐users require lifelong care, both the rehabilitation and research team were concerned that if service‐users were identifiable, they might be hesitant to fully disclose their experiences or concerns, particularly if they feared future discrimination or unequal treatment. Consistent with protecting service‐users' confidentiality and anonymity, we (i.e., research team) did not bring service users (i.e., service‐users and caregivers) and service‐providers together to formulate the priorities for action. Instead, priorities were communicated with each group individually through the medium of an independent researcher. Details of each step of the EBCD process are further outlined below. For an overview of the process, please see Figure 1.

### 2.2 Setting and Context

This research took place at a UK–based amputee rehabilitation centre operated by the NHS. The rehabilitation centre is in a community hospital but operates as a relatively self‐contained unit comprising a medical clinic, rehabilitation gym, prosthetic workshop, and consultation rooms. The multidisciplinary team is comprised of rehabilitation consultants, prosthetists, orthotists, physiotherapists, occupational therapists (OTs), clinical psychologists, nurses, social workers, and gait lab specialists. Service‐users attending the centre have undergone amputation due to a variety of causes, including vascular conditions, trauma, infection, disease or congenital defects. Service‐users can attend the rehabilitation centre full‐time, either as an in‐patient (due to complex needs) or an out‐patient for multiple weeks post‐surgery. Rehabilitation involves group classes (e.g., exercise and balance classes), educational talks (e.g., falls and pain management), and personalised rehabilitative exercises (e.g., learning how to use a prosthetic leg).

#### 2.2.1 Phase 1: Setting up the Project

An outside‐in pathway [21] was used to form a working group between the limb centre, service‐users, caregivers, and researchers to identify and address gaps in transitional care. The research team consisted of the first author, who has experience in qualitative research and a background in physiotherapy but is considered an ‘outsider’ within the amputee population. The second and third authors have expertise in co‐design and a long‐term relationship with the limb‐centre through previous research projects. The fourth author has experience in qualitative research and the psychology of injury. Following university and NHS ethical board approval, maximum variation purposeful sampling [22] was used to engage multiple voices and perspectives. Ten staff members (physiotherapists, OTs, rehabilitative assistant, clinical psychologist) were recruited (nine females and one male). Fourteen people with limb loss (see Table 1 for demographics) and 15 nominated caregivers were also recruited. This sample size was based upon previous research conducted with this population [5, 9, 12]. To ensure diverse perspectives, several criteria were used. Gender was considered by including both male and female participants, given that the experience of an amputation can differ across genders and that caregivers' roles can be gendered. Different types of amputations were included to capture the varying challenges for both service‐users and caregivers. To extend previous research, which has been largely limited to 1‐year post‐discharge, time since amputation was also considered to help explore the cumulative impact of caregiving. A range of caregiver roles—such as wife, partner, daughter and aunt—were included rather than focusing on a single role. Finally, a diverse range of practitioners were chosen (e.g., OT, physiotherapist and clinical psychologist). Together, these criteria ensured a broad range of perspectives. Information‐rich participants were identified through purposive sampling by members of the multidisciplinary team and the first author, who had been embedded within the service setting for 9 months prior to data collection. Information about the study was also disseminated to service users during follow‐up consultations within the rehabilitation clinic, providing an opportunity for self‐referral. Potential participants were subsequently contacted via email and provided with a participant information leaflet outlining the study. Two individuals who were approached declined participation.

**Table 1:** Participant characteristics and involvement.

Service‐user	Biological sex	Age range	Presentation	Trauma versus non‐trauma	Time since amputation	Nominated caregiver	Individual interview	Dyadic Interview	Working group (Service‐user only)
Heather	Female	45–50	Quadruple limb absence	Non‐trauma	4 years	Husband (Bill)	Yes	Yes	No
Anna	Female	25–30	Double‐leg limb absence	Non‐trauma	4 years	Partner (Jack)	Yes	Yes	Yes
Leslie	Female	40–45	Double‐leg limb absence	Trauma	6 months	Husband (Jotham)	Yes	Yes	No
Avery	Male	70–75	Double‐leg limb absence	Non‐trauma	2 years	Wife (Jane)	Yes	No	No
Dan	Male	55–60	Single‐leg limb absence	Non‐trauma	2 years	Wife (Lisa)	Yes	Yes	No
Jamie	Male	50–55	Single‐leg limb absence	Non‐trauma	18 months	Wife (Tina)	Yes	No	No
Rory	Male	45–50	Single‐leg limb absence	Non‐trauma	6 months	Partner (Ailbhe)	Yes	Yes	Yes
Theo	Male	45–50	Single‐leg limb absence	Trauma	1 year	No caregiver (lives alone)	Yes	No	No
Simon	Male	50–55	Double‐leg limb absence	Trauma	4 years	Wife (Sara)	Yes	Yes	No
Christine	Female	40–45	Quadruple limb absence	Non‐trauma	3 years	Aunt (Cara)	Yes	Yes	No
Micah	Female	80–85	Double‐leg limb absence	Non‐trauma	10 years	Husband (Andrew)	Yes	Yes	No
Sascha	Female	50–55	Quadruple limb absence	Non‐Trauma	4 years	Daughter (Jamie)	Yes	No	No
Dana	Female	40–45	Single‐leg limb absence	Non‐trauma	7 years	Husband and 2 children (John, Mia, and Andrew)	Yes	Yes	Yes
Lee	Female	20–25	Double‐leg limb absence	Trauma	4 years	Dad (Rhys)	Yes	No	Yes

Fourteen service users were recruited for the study. Each was invited to nominate a caregiver to participate, although one service user chose not to nominate a caregiver. All service users and nominated caregivers completed individual interviews. Following these interviews, nine service users and nine caregivers agreed to take part in a subsequent dyadic interview. Participants who declined to participate in the dyadic interview cited caregiver time constraints as the primary reason. In addition, all service users and caregivers were invited to take part in the co‐design working groups, of whom four service users agreed to take part. Details of the participants' involvement in the study are further outlined below.

#### 2.2.2 Phase 2: Gathering Experiences

The study design was underpinned by interpretivism, that is, ontological relativism (i.e., reality is multiple, created and mind‐dependent) and epistemological constructionism (i.e., knowledge is constructed and subjective) [23]. In line with this overarching philosophy and the commitment of EBCD to phenomenology, generating in‐depth insights into service‐users, providers, and caregivers' experiences of the service was central to both the design and execution of the project. Indeed, the authors of EBCD advocate for ethnographic paradigms and contextual enquiry, highlighting how 'without these methods, what often poses as experience research is actually little more than a conversation that anyone might have had, and words and stories without analytical frameworks do not speak for themselves' [24]. Therefore, although ethnographic observation is omitted in 80% of ECBD studies [19] we viewed it as being integral to generating contextualised understandings of the rehabilitation environment and participants' lived experiences.

Becoming embedded in the rehabilitation centre on a weekly basis for 8 h a week for 16 months (*n* = 512 h total), and shadowing the OT during home visits, ensured that the first author remained committed to the four principles (i.e., immersive and emic understanding, reflexivity, empathy) for exploring and understanding service‐user and provider experiences in EBCD [25]. For example, *immersive* and *emic* (insider) understandings were generated by observing the day‐to‐day processes of the rehabilitation setting, alongside observing how the OTs prepared service‐users and families for the transition during home visits. Such insights helped furnish an understanding of how and why things work (or not), and how they might be redesigned [25]. By taking field notes of her observations and informal conversations, the first author was also able to engage in *reflexivity* and consider how her own biases and assumptions may influence the data collection process. To do this, the first author kept a reflexive journal and engaged in processes of both introspective reflexivity (i.e., a process of using self‐understanding for interpretations and general insight) and intersubjective reflexivity (i.e., a process of reflecting on the researcher concerning the participants) [26]. For example, the first author reflected upon how her own background (physiotherapy), had led her to assume that the level of impairment would influence an individual's level of function and well‐being. However, this extensive period of observation mitigated that assumption as she observed how a myriad of factors (socio‐economic status, social support, cultural differences) influenced an individual's perception of their impairment, well‐being, and function. To further develop these reflexive processes, the first author shared her reflections and interpretations of the data regularly with the other members of the research team who encouraged reflection upon, and exploration of, alternative explanations and interpretations [27]. Finally, the ethnographic observation ensured fidelity to *empathetic understandings* by observing, listening, and inquiring; the first author was able to gain nuanced understandings into what it was like to be cared for and to provide care within this rehabilitation setting [25].

### 2.3 Staff Interviews

Alongside the ongoing ethnographic observation and aligned with the recommended phases of co‐design, semi‐structured interviews were conducted with staff to gain insights into their experiences of transitional care. Staff members were interviewed first due to their experiential knowledge of the transition process and wide breadth of experience, which helped develop the context for the latter interviews with service‐users and caregivers. Ten staff members were interviewed in person. Interviews lasted between 45 and 75 minutes, were recorded, and transcribed verbatim.

### 2.4 Service‐User and Caregiver Interviews

Following interviews with staff, semi‐structured interviews were conducted individually with service‐users (*n* = 14), then their nominated caregivers (*n* = 15), and finally dyadically (service‐user and caregiver *n* = 9) to help understand both their individual and collective experiences. For demographic details, please see Table one. Interviews were conducted either in‐person at the rehabilitative centre, online, or by telephone, depending on participants' preferences (see Appendix S1 for interview guide). Interviews lasted between 45 and 70 minutes, were recorded, and transcribed verbatim. In total, 3143 minutes of interview data were recorded.

#### 2.4.1 Phase 3 Data Analysis and Findings

To analyse the staff interviews, a six‐stage reflexive thematic analysis [28] was used. This involved the first author initially familiarising herself with the data by reading and re‐reading the transcripts. Preliminary codes were created by the first author and collated into a spreadsheet on Microsoft Excel. These codes were then shared with the rest of the research team and further discussed before clustering them together around a central organising concept to form overarching themes. These themes were then reviewed against the transcripts and the entire data set. The themes were named to capture interpretative stories of the data, and finally, they were written up.

Aligned with underpinning methodology of EBCD, a thematic narrative analysis [29] was used to analyse the service‐user and supporter interviews. A thematic narrative analysis involved identifying patterns across the data set that related to the content of participants' stories. The thematic narrative analysis focused on understanding the experiences and challenges faced by service‐users and their caregivers during the transition period as well as the impact of the amputation on their lives and relationships. The individual interviews were analysed first to help understand their individual experiences of the transition, before consulting the dyadic interviews to help furnish greater insights into their collective experiences. Following a period of indwelling (i.e., reading and re‐reading the transcripts), forming analytical notes and codes, narrative themes were identified. These themes included challenges faced by service‐users (e.g., body image, social stigma) and caregivers (burden of caring, feeling unprepared for the role), identity changes, negotiating changes within the relationship and in their own roles and responsibilities. A structural analysis was then used to identify the underlying plot of participants' stories. For example, we identified the various trajectories of service‐users post‐amputation. While some service‐users experienced an initial decline in well‐being and function post amputation; this was then followed by a subsequent increase in well‐being and function over time as the service‐user began to reclaim some of their former roles and identities. This type of plot coincided with an 'adaptation' narrative. Once the plotline was developed, we began to map the service‐users' stories and themes onto their various trajectories, using the participant quotations as much as possible to help convey the emotive and authentic elements of the stories. By framing participants' quotations within a narrative structure, we aimed to *show, not tell* their experiences in a compelling and evocative manner.

While the narratives from the caregivers and service‐users were used during the dissemination events to engage service‐providers with lived experiences and to help foreground touchpoints (i.e., crucial moments that make a difference to someone's experiences of the environment or process) [24], an inductive content analysis was undertaken to systematically identify gaps in transitional support and potential solutions. A content analysis was chosen as it provides a systematic method for analysing and ordering practical suggestions for change and thus helps make the data more manageable and actionable by grouping it into sub‐categories of support gaps and recommendations [30]. Responses from service‐users, caregivers, and healthcare professionals to interview questions relating to experiences of transition from hospital to home, unmet support needs, and recommendations for improving transitional care were collated and analysed collectively. Using an inductive approach, responses were coded, compared, and grouped according to similarities in meaning. Codes were subsequently organised into subcategories representing common support gaps and recommended service improvements. These findings informed discussion and priority setting during the co‐design process.

### 2.5 Methodological Rigour

In line with our overarching philosophy (i.e., interpretivism), a relativist position was used to enhance the methodological rigour and trustworthiness of the study [31]. The rigour of the research was attended to by selecting an information‐rich sample, the use of maximum‐variation sampling to enhance the study's generalisability, including multiple perspectives to enhance the richness and robustness of the findings, alongside prolonged data collection and engagement in the field [32]. Moreover, the sincerity and trustworthiness of the findings were attended to through self‐reflexivity [26], where the first author engaged in reflexive journaling, reflecting on her biases and assumptions and how they may have influenced the data analysis, and critical friend discussions [27] with the rest of the research team, where alternative interpretations were sought. To further enhance the credibility of the findings external reflections were sought by sharing the findings with the staff regularly during in‐service events, and also with service‐users involved in the working groups.

To maintain the overall rigour and methodological quality of the project, we also remained faithful to the four core principles of EBCD throughout [13]. These include a *participatory focus*, as we aimed to ensure that this was a collaborative process that included multiple stakeholders' views (i.e., service‐users, caregivers, HCPs, and the wider medical team), which were elicited over time. *Ownership and power* involve dismantling hierarchical relationships between staff and service‐users and ensuring that service users and caregivers are active partners in the design of their care. To this end, we aimed to use service‐users' and caregivers' narratives to create shared understandings, protect service‐users' and caregivers' confidentiality to ensure that their feedback was uncensored [20] and attend to the recommendations suggested by service‐users and caregivers as well as actively involve them in co‐addressing these recommendations. *Development as* a core principle was attended to by honouring the cyclic reflective cycle associated with co‐design, including making adaptations as the project progressed. Finally, *outcomes and intent* were attended to by ensuring that the co‐design process had a practical focus, and the resources and action points co‐created could be readily implemented into practice.

## 3 Findings

Overall, the findings were divided into two parts: Understanding the transition experience and recommendations for bridging the gap. A summary of the main findings are outlined below.

### 3.1 Part One: Understanding the Transition Experience

#### 3.1.1 HCPs Perspectives

Four themes were identified from the HCP data which highlight the challenges associated with the transition experience. The first theme, ‘We can't replicate home life here’ reflected HCPs struggles in replicating tasks that service‐users may encounter at home. The second theme ‘Stretched resources’ spoke to practitioners' concerns over their own time limitations which precluded them from engaging in community‐based rehabilitation with service‐users. Additionally, this theme relates to stretched resources within the community, leading to poor transitional care, due to long waiting lists, a de‐prioritisation of limb loss service‐users, and limited training of community staff. The third theme, ‘It's a life‐changing experience’ related to HCPs perspectives on the experiences and challenges that service‐users and their caregivers may face following amputation. The fourth theme, ‘Limited insights’ related to HCPs' perceptions of the gaps in communication between them and caregivers.

#### 3.1.2 Service‐Users and Caregivers' Perspectives

**Figure 2:**
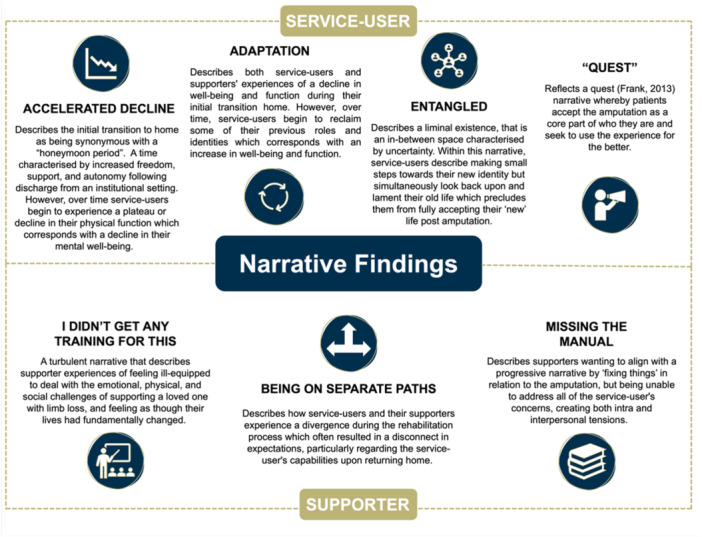
Narratives of service‐users and caregivers.

Overall, we constructed four narratives which described the trajectories of service‐users post‐amputation, and three narratives which narrated the impact of amputation on their lives and relationships from a caregiver's perspective. These narratives are represented in Figure 2.

### 3.2 Part Two: Recommendations for Bridging the Gap

Service‐users, caregivers, and HCPs identified four gaps in transitional care. First, an information support gap was identified. While service‐users acknowledged receiving information through educational sessions during rehabilitation, many described difficulty recalling or applying this information once they returned home, as they were simultaneously adjusting to the physical, emotional and practical realities of life with limb loss. Caregivers similarly reported unmet informational needs, noting that they were often not present for educational sessions and therefore had limited access to information regarding rehabilitation, practical care needs, safety considerations, financial supports and long‐term adjustment. HCPs highlighted how although service‐users are provided with educational talks, follow‐up telephone support, and written materials upon discharge, they often noted informational gaps during follow‐up consults and highlighted how existing resources may not adequately reflect the complexity of service‐users' and caregivers' needs. Staff highlighted the need to update educational materials to improve alignment with information delivered during rehabilitation and to provide a resource that could serve as a reference manual for both service‐users and caregivers.

**Figure 3:**
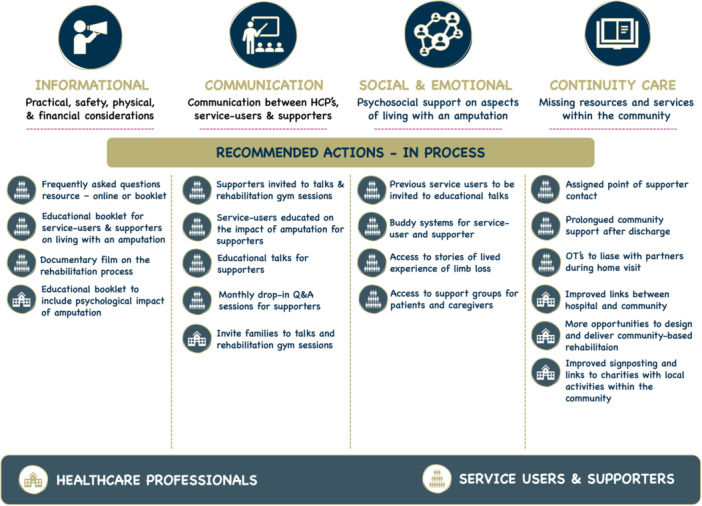
Recommendations for bridging the gap.

Second, gaps in emotional and social support were identified; all participants highlighted how service‐users could benefit from having greater information about the psychosocial challenges of living with an amputation and connection to previous service‐users and caregivers who had lived experiences of such challenges. Third, gaps in communication between the HCP's and the service‐users' caregivers were identified. Caregivers reported having limited insight into the rehabilitation process leading to a mismatch in expectations, concerning the service‐users' functional abilities, upon returning home. HCPs highlighted how although they invite caregivers to the rehabilitation gym, given the patient centred ethos of the NHS, they are led by the preferences of the service‐users creating challenging in balancing patient autonomy and caregiver involvement. That said, HCPs highlighted the need for more formalised and proactive approaches to including caregivers throughout the rehabilitation process to address their educational, communication, and support needs. Finally, gaps in care continuity, including access to community services such as OT and physiotherapy and linking to relevant resources within the community were highlighted. Recommendations for addressing these gaps were further outlined by service‐ users, providers and caregivers and detailed in Figure 3.

#### 3.2.1 Phase 4: Sharing Experiences

Aligned with the fourth stage of EBCD [24], we aimed to share service‐users' and caregivers' experiences of the service and identify and action change. Three dissemination events took place, one with service‐user participants, one with the immediate rehabilitation team and one with the wider medical team.

### 3.3 Service‐User Dissemination Event

The service‐user dissemination event involved a focus group with four service‐users. Within this focus group, the findings from the study (i.e., caregiver and service‐user narratives) were discussed alongside the four support gaps (see Figure 3) and associated recommendations for change. Discussions centred around the importance of certain support gaps and action points as well as highlighting additional recommendations for change. These included (1) the need for access to information earlier in the rehabilitation process (i.e., during intake meetings), (2) the need for an updated educational booklet in a more durable format that should be referenced throughout the rehabilitation talks, (3) more opportunities to share stories within educational talks and (4) an additional talk to be delivered concerning the psychosocial aspects of living and supporting someone with an amputation. For example, Lee describes:

“I think after amputation, finding your new normal is still very much a progressive thing that changes all the time, so certainly having a booklet can be useful as a reference manual that you might refer to at those different stages…but I think including stuff in the booklet around psychological aspects is great, but that also needs to be covered in talks too, because although there was a lot of practical stuff around falls, we didn't really cover much on the psychological side of things.”

These additional action points were added to the previous collated list and then shared in dissemination events with staff.

#### 3.3.1 Staff Dissemination Event

Alongside the ongoing feedback to staff through informal conversations and monthly CPD days, there were two dissemination events with both the immediate and then the wider rehabilitation and medical team. Within the dissemination events, the seven service‐user and caregiver narratives were shared and discussed with staff members. Narratives are viewed as key resources that provide a way for HCPs to emotionally connect with service‐users' and caregivers' experiences, thus mobilising them towards reflection and change [17]. Narratives are also dialogical [33], and within these dissemination events, they created discussions around the psycho‐social implications of an amputation, as well as the impact of amputation on caregivers, whom they acknowledged were often the forgotten voice within rehabilitation settings.

Following these discussions, the four support gaps (see Figure 3) were considered in turn alongside service‐users' and caregivers' recommendations for how they could be addressed. To guide these discussions, we drew upon the APEASE criteria to determine the feasibility of these action points by considering the Affordability, Practicability, Effectiveness, Acceptability, Safety and Equity components in line with evidence‐based recommendations for implementing change [34]. Moreover, while we aimed to keep the action points grounded in service‐users, caregivers, and HCPs recommendations, the research team further consulted the literature to ensure that changes prioritised were evidence‐based.

To address the *information gap* and in line with service‐user, caregiver, and HCPs recommendations, it was decided that a new updated format of the educational booklet would be co‐designed and created (see next section for more details). Co‐designing educational material with rehabilitation staff, service‐users, and caregivers has proven effective in other chronic conditions such as stroke management [35]. This success has been attributed to the capacity of educational material, in the form of booklets with appropriate personalisation for service‐users, to provide a greater understanding of the post‐rehabilitation period and promote person‐centred care by enabling service‐users to tailor their management approaches accordingly [36].

To address *gaps in communication* between HCPs and caregivers, it was agreed that greater efforts would be made to invite families to the rehabilitation gym and educational talks. The rehabilitation and wider medical team acknowledged that while they often try to include family members, a more formalised approach is needed. Developing a drop‐in Q&A session for caregivers in line with caregivers' recommendations, was put forward as an action point for the rehabilitation team to address.

Centralising service‐users and caregivers lived experiences both within the educational booklet and by inviting previous service‐users back to educational talks was agreed by all HCPs as being imperative in addressing *gaps in emotional and social support*. As Frank (2010) depicts, people need to hear their own stories, and by hearing their own stories become empowered to tell their own [37]. Giving primacy to storytelling within educational talks and material, can therefore help support service‐users and caregivers' emotional well‐being, by normalising and validating their experiences, creating a sense of solidarity, whilst also offering them the space and opportunity to make sense of their own lived experiences [33]. To further enhance social support, the staff discussed reforming links with the currently lapsed user group, as well as signposting to local buddy schemes and charities within the community. In line with service‐users' and caregivers' suggestions and following on from conversations concerning the psycho‐social aspects of living and supporting someone with limb loss, it was decided that an additional talk would be added to the weekly educational series, delivered by the clinical psychologist, and covering issues foregrounded by participants such as body image, self‐esteem and mental well‐being.

Finally, *gaps in care continuity*, although acknowledged as being pertinent in bridging the gap between hospital to home, were viewed as the most difficult to address using the APEASE criteria [34]. For example, service‐users, caregivers, and HCPs all acknowledged the need for service‐users and caregivers to have access to greater therapy support within the community once discharged from rehabilitation. That said, as highlighted within staff interviews, access to this support was difficult, due to long waiting lists, a de‐prioritisation of limb loss service‐users unless they are at falls risk, and inadequate resourcing and training of community OT and physiotherapy staff. These issues are reflective of wider healthcare issues and staff shortages within the NHS more generally. However, to address these concerns, practitioners suggested greater signposting to activities within service‐users' local communities, advising service‐users and caregivers that they can call the rehabilitation centre at any stage with any questions, and inviting community physiotherapists and OTs to their in‐service training days to enhance their knowledge regarding amputation management.

**Figure 4:**
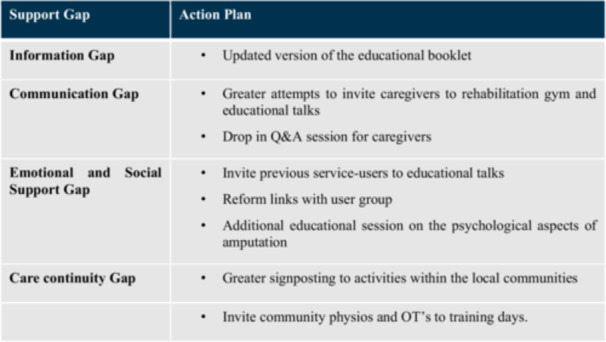
Support gaps and action plan.

Following these discussions, recommendations and action points were emailed to all service‐users and caregivers for further feedback (see Figure 4). Following further consultation with the service‐user working group, it was decided that the project would focus on co‐designing the educational booklet for service users and caregivers. In line with the APEASE criteria, the booklet was deemed the top priority, as it could be actioned and implemented immediately. Moreover, developing an educational booklet for both service‐users and caregivers helped address all four support gaps, by providing information, addressing communication and care continuity gaps by signposting service‐users and caregivers to resources in the community, and addressing social and emotional support gaps by using stories of caregivers and service‐users lived experiences, and signposting to resources related to mental well‐being.

#### 3.3.2 Phase 5: Co‐Designing Resources for Impact

The development of the booklet was led by the primary researcher in collaboration with the rehabilitation team, service users, and caregivers. To facilitate the construction of the booklet, three working groups were established. These included a working group with the first author and HCPs, a working group between the HCPs and three current in‐patients, and a working group between the first author and service‐users.

### 3.4 Construction of the Booklet

To help develop the informational material, the first author first reviewed all of the educational material created and provided by the rehabilitation clinic. Moreover, the first author attended all seven educational talks delivered by the rehabilitation centre on multiple occasions. By attending these talks, she aimed to create coherence between the information discussed during rehabilitation and the booklet, in the hope that this may bridge the gap that can sometimes appear between the real‐world and rehabilitation. Following this review period, the first author documented the information currently available (in the form of educational material), gaps in informational support (based on service‐user and caregiver interviews), as well as feedback from service‐users with regard to the original educational material. Any gaps in information were then co‐created between the first author and relevant staff members. For example, information about financial benefits was co‐created between the first author and social worker, whilst information about psychological support was co‐created with the clinical psychologist.

Alongside this informational support, and consistent with service‐users' and caregivers' suggestions, we aimed to provide future users with insight into lived experiences of managing the hospital to home transition. Heeding recommendations from previous researchers, we aimed to ensure that our educational and interventional design had explicit theoretical underpinnings [35], by grounding it in narrative inquiry [37] and therapy [38]. Therefore, we included narratives of limb loss within the booklet (see Figure 2), as well as opportunities for users to construct their own story of limb loss. Alongside these overarching narratives, we also used smaller participant stories to forewarn users of potential challenges ahead and forearm them with possible solutions. Overall, stories were used to render the educational material more accessible and relatable to future users, provide orientation and guidance with regard to the journey ahead, and equip users with the tools to make sense of and understand their own limb loss journey. One service‐user described:

“Having the stories there is really powerful, because it just helps prepare you, and also just gets the ball rolling in terms of these are things you might be feeling, and you're not alone in feeling like that…and then the exercises are something that you can come back to if you do feel a bit stuck, so it just gives you those building blocks to start to having a conversation about these things.”

The booklet went through multiple iterations based upon feedback from the three working groups. This feedback was used to improve the readability, user‐centeredness, and implementation of the booklet. The final version of the booklet is 104 pages in total and includes six different sections: Living with an Amputation, Exercise and Staying Active; Looking after you: The Psychological Side of an Amputation; Relationships and Amputation: Support for the Caregiver; Physical Relationships and Sexual Lifestyle Following Amputation; Looking Back to Look Forward: Tools to Help Navigate the Journey. Currently, the booklet has been embedded in practice within the rehabilitation centre.

## 4 Discussion

Heeding calls to address gaps in transitional care for people with limb loss and their caregivers, the aim of this study was to (a) understand the transition from hospital to home and identify gaps in transitional care; and (b) co‐construct a resource that aims to bridge these gaps and better facilitate the transitionary process. Previous research has highlighted how gaps in care continuity, including informational, emotional, and social support, can leave people with limb loss and their caregivers feeling ill‐equipped to navigate this transition [5]. Ultimately, this lack of readiness to return home can result in hospital readmissions, further complications, and increased healthcare costs [11, 12]. To address these aforementioned gaps in transitional care, this study employed an EBCD approach. Building on previous research that conducted research ‘on’ rather than ‘with’ participants (i.e., service‐users, caregivers and hospital staff), we aimed to engage participants as active collaborators in identifying current gaps in transitional care and in addressing these gaps by co‐creating action points and resources for change. This approach works symbiotically with the person‐centred care ethos of healthcare policies and initiatives by ensuring that the voices and experiences of service‐users and their caregivers are foundational in the development of evidence‐based strategies to drive and create meaningful and sustainable change.

Building upon previous research, which has predominately explored the perspectives of healthcare workers [12], service‐users, and caregivers either in isolation or dyadically [1, 2, 3, 5, 9], this study extends the literature by integrating all three perspectives. Previous research with HCPs has identified patient preparedness, follow‐up support, funding constraints, self‐management skills, psychosocial support and communication across the continuum of care as important influences on successful transitions [12]. Similarly, research involving individuals with limb loss have highlighted the need for improved access to information, psychological support, coordinated rehabilitation services and ongoing community‐based follow‐up [1, 5, 9]. Studies involving caregivers have further emphasised the importance of educational support, inclusion in discharge planning, and recognition of caregivers as active partners in the rehabilitation [10]. Likewise, our study identified four gaps in transitional support, including information‐support gaps, care continuity gaps, emotional and social support gaps and gaps in communication between HCPs and caregivers. However, by integrating the perspectives of HCPs, service‐users, and caregivers, this study extends the literature by providing a more holistic, nuanced and comprehensive understanding of the key gaps in transitional care. For example, while caregivers and service‐users identified gaps in informational support, HCPs were able to provide greater insight and context into the nature of this gap by highlighting how it was not a lack of information per se but rather how the information was being delivered, emphasising the need for updated educational resources. Such insights emphasise the benefit of EBCD in ensuring that interventions developed to improve transitional care are contextually grounded and relevant for the setting.

Towards this end, this research extends previous work by moving beyond the identification of gaps in care towards the co‐creation of recommendations for service improvement. Drawing upon EBCD, stakeholders were actively involved in developing solutions to the challenges identified, ensuring that proposed changes reflected the priorities and experiences of those directly involved in the transition process [17]. The incorporation of the APEASE criteria [34] further strengthened this process by ensuring that recommendations were grounded within the realities of rehabilitation service delivery. For example, although enhanced care coordination has been consistently highlighted within the literature as a key mechanism for improving transitions [9, 12], participants identified substantial organisational and funding barriers that limited the ability of staff to address this issue directly. In contrast, the development of an educational booklet emerged as a practical and implementable intervention capable of addressing multiple support gaps simultaneously. Therefore, this study provides novel insights into the feasibility of different improvement strategies.

Finally, while previous studies have advocated for greater involvement of caregivers as partners in rehabilitation [39], limited attention has been given to how this can be operationalised in practice. By actively incorporating caregivers' experiences, perspectives, and support needs into the educational resource, this study extends current knowledge by demonstrating a tangible mechanism through which caregivers can be recognised, supported, and integrated within transitional rehabilitation pathways. As such, the study not only advances understanding of transitional care needs following limb loss but also provides practical, stakeholder‐informed strategies, to support service development and implementation.

### 4.1 Strengths and Limitations

A key strength of this work was the use of EBCD, which enabled end‐users to play an active role in identifying gaps in support and co‐designing solution‐oriented recommendations. This collaborative approach ensured that the outcomes were not only relevant to users' needs but also practical, and hence they could be readily implemented into practice. Embedding the primary researcher within the setting for 18 months further strengthened the study. It enabled her to develop nuanced and contextualised understandings of the setting alongside end‐users lived experiences, which guided both the design of the study and the implementation of recommendations. The extensive data collection and inclusion of multiple stakeholder groups further contributed to a robust and comprehensive understanding of the issues at hand. Finally, a key strength was the co‐creation of the educational booklet. This resource addressed all four identified support gaps and has already been implemented into practice, demonstrating its immediate applicability. Importantly, while the booklet was co‐designed with stakeholders, it was also underpinned by relevant theory [37, 38] and informed by the existing evidence base. As such, it represents both a user‐centred and evidence‐based intervention, with potential for application beyond the current rehabilitation setting.

A key limitation of this work was that it was restricted to one rehabilitation centre, which potentially limits the transferability of the findings. Future research is needed to explore the transferability of these support gaps and recommendations to other rehabilitation contexts. That said, by detailing how we employed an EBCD process within a limb loss context, this study provides a blueprint and springboard for future researchers and limb centre rehabilitation teams to consider how they may address gaps in transitional care for both service‐users and their caregivers. Consistent with previous research conducted with vulnerable populations [20], we opted for a flexible adaptation of EBCD rather than a rigid approach. For example, to protect the anonymity of our participants, we chose not to film participants or bring service‐users and service‐providers together. As people with limb loss require lifelong care, such adaptations allowed us to ensure that service users felt comfortable to fully disclose their experiences and that they were protected against potentially unfair treatment in the future. That said, by not bringing service‐users and providers together, there is the potential that we missed the opportunity for more shared understandings and collaboration in the co‐design of solutions. Finally, due to time constraints, no caregivers were involved in the working group in relation to the co‐construction of the booklet. While feedback was obtained from some caregivers via email, this method limited their level of engagement in the co‐design process.

## 5 Conclusion

Overall, these findings extend the literature by outlining the current gaps in transitional care within a limb loss context based upon service‐users, caregivers, and HCPs perspectives. Moreover, these findings provide an evidence base for how to bridge these gaps through a co‐created resource (i.e., educational booklet) with service‐users, caregivers, and HCPs. Given the recent policy initiatives (e.g., UK 10 Year Health Plan [40]) aimed at transitioning service‐users from the acute settings to the community, alongside the ongoing impetus for person‐centred care, this evidence base is timely.

## Author Contributions

**Ciara Everard:** conceptualisation, investigation, funding acquisition, writing – original draft, methodology, writing – review and editing, formal analysis, project administration, data curation. **Melissa C. Day:** conceptualisation, funding acquisition, methodology, writing – review and editing, formal analysis, project administration, supervision. **Ross Wadey:** conceptualisation, funding acquisition, methodology, writing – review and editing, formal analysis, supervision. **Kimberley Humphrey:** methodology, visualisation, writing – review and editing, formal analysis, project administration, data curation, supervision, resources.

## Ethics Statement

Ethical approval was received by the NHS ethical approval board and University of Chichester approval board. Participants provided informed consent.

## Conflicts of Interest

The authors declare no conflicts of interest.

## Supporting information


Supporting File 1

Supporting File 2

Supporting File 3

Supporting File 4

## Data Availability

The data that support the findings of this study are available on request from the corresponding author. The data are not publicly available due to privacy or ethical restrictions. Participants of this study did not agree for their data to be shared publicly, therefore, supporting data is not available.
