# Supplementary material for: Bridging the Gap: Developing a Co‐Designed Resource to Support the Hospital‐to‐Home Transition Following Limb Loss
Source: Health Expect. 2026 Aug 17;29(4):e70825. doi: 10.1111/hex.70825 (PMC13482350; doi:10.1111/hex.70825)
Supplement: Supplementary file 1 [file HEX-29-e70825-s001.docx]

**Interview with Patient who does not want to nominate a support provider**

Welcome (general welcome used for all interview types)

*Introduction and overview*

- Introduction of researcher and project aims
- Overview of plan for study:

o An interview lasting no more than one hour which will be recorded, explanation regarding data handling and anonymisation of transcripts.

o Feedback on results: Results presented as a written overview, individual will be provided with a copy and asked to provide any feedback (by email or telephone).

- Review of PIS and completion of written consent form
- “Feel free to ask questions at any stage”

We will now move on to our interview questions which explore the transition from hospital to home.

**Planning for the transition**

In looking specifically at this transition process

- Firstly – when did you start thinking about going home?
- Who prepared you for that experience?
- What information did you receive?
- How did you each feel about the return home?
- Was there anything that concerned you?
- Did it change over time (initial excitement – reality)
- Can you give me an example/story that illustrates your experiences?
- Any particular experiences that stand out for you? Why?

**Experiencing the transition**

Can I ask you to now think about when you did return home.

- How was the experience in comparison to your expectations?
- Did you experience any challenges when you returned home? [talk through each challenge described – how it was experienced]
- When you returned home – what were you able to do around the home?
- Is this different to before?
- How so?
- How did this impact you?
- What adjustments were made to your home environment?

**Support received from others**

- Did anyone else provide support for you?
- If so, can you tell me a bit about how they have supported you? [talk through any other support providers in turn]
- Are there any support needs that you feel have been unmet?
- Did you experience any challenges in gaining support from others?

**Transition from hospital to home**

In terms of the hospital to home transition, do you see any gaps in the service at present?

- Describe in greater detail? Give me an example/story about this?
- How would you address these gaps/ how should they be addressed?

**Life after returning home**

- Did you feel like you were adequately prepared for life after rehabilitation?
- How so?
- What was done well/not so well
- What information do you feel like you needed prior to discharge?
- What information/advice was useful?
- Did you use the booklet that was given to you at the beginning of rehab? Did you find this useful? If you didn’t use it, why not? How do you think it could be improved?
- Was there any support you felt you needed but didn’t receive?
- What would you change if anything?

**Post-Discharge**

- What information did you want access to?
- How did you get access to this information?
- Was it readily available?
- Any other support/resources you felt like you needed? Did you get access to this? How so? Was it helpful?
- Have you had support from any others who have experienced lower limb loss since leaving hospital – has this helped/not helped?

**Resources at Present**

As part of this project, we are looking to develop resources to better facilitate the hospital to home transition for patients and their supporters…

- What resources/support do you think you need/needed to help better facilitate this transition? (e.g., stories of other who have been through it, information book with all relevant information)
- Prompts – Why? How do you think that might help?
- Tease out across the different phases - admission, inpatient, and return home?
- Do you think you needed different resources/support at different phases of the transition?
- What format do you think is best to deliver these resources?
- At what stage should these resources be delivered?

**Feedback**

- Provide opportunity for any questions or further discussion
- Provide participant with reminder information:
- If you wish to follow up any issues you have talked about, you can contact me (reminder of contact details)
- Right to withdraw data
- Overview of results will be sent to them to provide feedback.

Thank participant for their time.
