# Supplementary material for: Bridging the Gap: Developing a Co‐Designed Resource to Support the Hospital‐to‐Home Transition Following Limb Loss
Source: Health Expect. 2026 Aug 17;29(4):e70825. doi: 10.1111/hex.70825 (PMC13482350; doi:10.1111/hex.70825)
Supplement: Supplementary file 2 [file HEX-29-e70825-s002.docx]

**Topic Guide for Dyadic Interview with Patient and Support Provider**

Welcome (general welcome used for all interview types)

*Introduction and overview*

- Introduction of researcher and project aims
- Overview of plan for study:

o An interview lasting no more than one hour which will be recorded, explanation regarding data handling and anonymisation of transcripts.

o Feedback on results: Results presented as a written overview, individual will be provided with a copy and asked to provide any feedback (by email or telephone).

- Review of PIS and completion of written consent form
- “Feel free to ask questions at any stage”

We will now move on to our interview questions which explore the transition from hospital to home.

**Planning for the transition**

In looking specifically at this transition process

- Firstly – when did you start thinking about going home?
- Who prepared you for that experience?
- What information did you each receive?
- How did you each feel about the return home?
- Was there anything that concerned you?
- Did it change over time (initial excitement – reality)
- Can you give me an example/story that illustrates your experiences?
- Any particular experiences that stand out for you? Why?

**Experiencing the transition**

Can I ask you to now think about when you did return home.

- How was the experience in comparison to your expectations?
- Did you experience any challenges when you returned home? [talk through each challenge described – how it was experienced/supported]
- When you returned home – what were you able to do around the home?
- Is this different to before?
- How so?
- How did this impact each of you?
- What support was needed?
- Did you feel able/equipped to give this support?
- What adjustments were made to your home environment?

**Life after returning home/Resources**

- Is it as expected? How so? If it’s different, how so?
- How have you found the experience overall as a collective?
- What do you perceive to be a ‘successful’ transition
- What support did you feel like you needed?
- What do you see as the main gaps in support from hospital to home?
- Is there any information that might have been valuable that you did not receive or any resources that you would have liked to have been provided with?
- What might have helped as someone who provides support?
- What might have helped as an individual going through the transition?
- What might have helped from a relationship perspective?
- Are there any resources that you feel would be useful for you both?
- Did you think that different resources are needed for each of you? If so, how so?

**Feedback**

- Provide opportunity for any questions or further discussion
- Provide participant with reminder information:
- If you wish to follow up any issues you have talked about, you can contact me (reminder of contact details)
- Right to withdraw data
- Overview of results will be sent to them to provide feedback.

Thank participant for their time.
