# Supplementary material for: Bridging the Gap: Developing a Co‐Designed Resource to Support the Hospital‐to‐Home Transition Following Limb Loss
Source: Health Expect. 2026 Aug 17;29(4):e70825. doi: 10.1111/hex.70825 (PMC13482350; doi:10.1111/hex.70825)
Supplement: Supplementary file 3 [file HEX-29-e70825-s003.docx]

**Topic Guide - Individual Interview with Support Provider**

Welcome (general welcome used for all interview types)

*Introduction and overview*

- Introduction of researcher and project aims
- Overview of plan for study:

o An interview lasting no more than one hour which will be recorded, explanation regarding data handling and anonymisation of transcripts.

o Feedback on results: Results presented as a written overview, individual will be provided with a copy and asked to provide any feedback (by email or telephone).

- Review of PIS and completion of written consent form
- “Feel free to ask questions at any stage”

Thank you for agreeing to talk with me. In this interview I would like to explore some of your personal experiences of supporting [patient] during the transition from hospital to home.

**Providing Support**

**Now we are going to talk about your experiences of providing support…**

- How was that experience for you as the support provider?
- Did you face any challenges? If so, can you explain these to me?
- Did you feel prepared for the transition?
- Who prepared you for it?
- What education/advice/information did you receive before [patient] returned home?
- How was the experience of transitioning back home? Did it change over time?
- Was the transition home as expected? If not, how so?
- In what ways have you supported them since they came back home? Has that role changed over time? How has that impacted you?
- What about your relationship to your home environment- has that changed since they returned home- if so, how?
- What have been the main challenges during this transition – so from leaving hospital to returning home? Have these challenges changed over time? (from when they initially returned home to now).

**Transition from hospital to home**

In terms of the hospital to home transition – do you see any gaps in the service at present?

- Describe in greater detail? Give me an example/story about this?
- How would you address these gaps/ how should they be addressed?

**Life after returning home**

- Did you use the booklet that was provided at discharge? Did you find this helpful? If so, why? If not, why not?
- Was there any information that you feel like you needed but didn’t receive?
- What sort of information/resources do you think you could have benefitted from to help facilitate the transition home and life after rehabilitation? (e.g., stories of others experiences, information about life at home, access to other caregivers?)
- Have you sought information from any other sources (e.g., websites, charities, other supporters?) if so what? Was it helpful?

**Resources at Present**

As part of this project, we are looking to develop resources to better facilitate the hospital to home transition for patients and their supporters…

- What resources/support do you think you need/needed to help better facilitate this transition? (e.g., stories of other who have been through it, information book with all relevant information)
- Prompts – Why? How do you think that might help?
- Tease out across the different phases - admission, inpatient, and return home?
- Do you think you needed different resources/support at different phases of the transition?
- What format do you think is best to deliver these resources?
- At what stage should these resources be delivered?

**Feedback**

- Provide opportunity for any questions or further discussion
- Provide participant with reminder information:
- If you wish to follow up any issues you have talked about, you can contact me (reminder of contact details)
- Right to withdraw data
- Overview of results will be sent to them to provide feedback.

Thank participant for their time.
