# Supplementary material for: Bridging the Gap: Developing a Co‐Designed Resource to Support the Hospital‐to‐Home Transition Following Limb Loss
Source: Health Expect. 2026 Aug 17;29(4):e70825. doi: 10.1111/hex.70825 (PMC13482350; doi:10.1111/hex.70825)
Supplement: Supplementary file 4 [file HEX-29-e70825-s004.docx]

**Topic Guide for Individual Interview with Patient**

Welcome (general welcome used for all interview types)

*Introduction and overview*

- Introduction of researcher and project aims
- Overview of plan for study:

o An interview lasting no more than one hour which will be recorded, explanation regarding data handling and anonymisation of transcripts.

o Feedback on results: Results presented as a written overview, individual will be provided with a copy and asked to provide any feedback (by email or telephone).

- Review of PIS and completion of written consent form
- “Feel free to ask questions at any stage”

Thank you for agreeing to talk with me. In this interview I would like to explore some of your personal experiences of the transition from hospital to home.

**Support received from others**

In relation to the support you received, as you mentioned you received support from (caregiver).

- Did anyone else provide support for you?
- Can you tell me a bit about how they have supported you? [talk through any other support providers in turn]
- Are there any support needs that you feel have been unmet?
- Did you experience any challenges in gaining support from others?
- Are there any experiences that you would like to talk about today that we didn’t pick up on in our last interview?
- Any experiences/stories that stand out to you?
- Looking back now, is there anything you wish your support providers had known or information/conversations you wish you had had with them?

**Transition from hospital to home**

In terms of the hospital to home transition, do you see any gaps in the service at present?

- Describe in greater detail? Give me an example/story about this?
- How would you address these gaps/ how should they be addressed?

**Life after returning home**

- Did you feel like you were adequately prepared for life after rehabilitation?
- How so?
- What was done well/not so well
- What information do you feel like you needed prior to discharge?
- What information/advice was useful?
- Did you use the booklet that was given to you at the beginning of rehab? Did you find this useful? If you didn’t use it, why not? How do you think it could be improved?
- Was there any support you felt you needed but didn’t receive?
- What would you change if anything?

**Post-Discharge**

- What information did you want access to?
- How did you get access to this information?
- Was it readily available?
- Any other support/resources you felt like you needed? Did you get access to this? How so? Was it helpful?
- Have you had support from any others who have experienced lower limb loss since leaving hospital – has this helped/not helped?

**Resources at Present**

As part of this project, we are looking to develop resources to better facilitate the hospital to home transition for patients and their supporters…

- What resources/support do you think you need/needed to help better facilitate this transition? (e.g., stories of other who have been through it, information book with all relevant information)
- Prompts – Why? How do you think that might help?
- Tease out across the different phases - admission, inpatient, and return home?
- Do you think you needed different resources/support at different phases of the transition?
- What format do you think is best to deliver these resources?
- At what stage should these resources be delivered?

**Resources for supporter/carer**

- How prepared do you think [support provider] was for the transition?
- What resources do you think your supporter needs/needed to help better facilitate this transition? Why? From who? What format

**Feedback**

- Provide opportunity for any questions or further discussion
- Provide participant with reminder information:
- If you wish to follow up any issues you have talked about, you can contact me (reminder of contact details)
- Right to withdraw data
- Overview of results will be sent to them to provide feedback.

Thank participant for their time.
